# The network of psychosocial health in middle-aged and older adults during the first COVID-19 lockdown

**DOI:** 10.1007/s00127-022-02308-9

**Published:** 2022-06-08

**Authors:** Maud de Feijter, Desana Kocevska, Tessa F. Blanken, Isabelle F. van der Velpen, M. Arfan Ikram, Annemarie I. Luik

**Affiliations:** 1 Department of Epidemiology, Erasmus MC University Medical Center, Rotterdam, The Netherlands; 2 Department of Child and Adolescent Psychiatry/Psychology, Erasmus MC University Medical Center, Rotterdam, The Netherlands; 3 Department of Sleep and Cognition, Netherlands Institute for Neuroscience, Amsterdam, The Netherlands; 4 Department of Psychological Methods, University of Amsterdam, Amsterdam, The Netherlands; 5 Department of Radiology and Nuclear Medicine, Erasmus University Medical Center, Rotterdam, The Netherlands

**Keywords:** Psychosocial health, Lockdown, COVID-19 pandemic, Network analyses, Middle-aged and elderly, Population-based

## Abstract

**Purpose:**

Psychosocial health problems, such as social isolation, loneliness, depression and anxiety, have gained attention during the COVID-19 pandemic and are commonly co-occurring. We investigated the network of psychosocial health constructs during the COVID-19 pandemic.

**Methods:**

This study included 4553 participants (mean age: 68.6 ± 11.2 years, 56% women) from the prospective Rotterdam Study, who filled out a questionnaire between April and July 2020, the time of the first COVID-19 wave in the Netherlands. Psychosocial health constructs included were depressive symptoms (Center for Epidemiological Studies Depression scale), anxiety symptoms (Hospital Anxiety and Depression scale), loneliness (University of California, Los Angeles loneliness scale), social connectedness (five items) and pandemic-related worry (five items). We estimated mixed graphical models to assess the network of items of these constructs and whether age and sex affected the network structure.

**Results:**

Within the network of psychosocial constructs, a higher depressive symptoms score was particularly associated with items of loneliness and social connectedness, whereas overall anxiety was particularly associated with items of pandemic-related worry. Between people from different sex and age, the network structure significantly altered.

**Conclusion:**

This study demonstrates that within the same network of psychosocial health constructs, depressive symptom score is particularly associated with loneliness and social connectedness, whereas anxiety symptom score is associated with pandemic-related worry during the first COVID-19 lockdown. Our results support that psychosocial constructs should be considered in conjunction with one another in prevention and treatment efforts in clinical care, and that these efforts need to be tailored to specific demographic groups.

**Supplementary Information:**

The online version contains supplementary material available at 10.1007/s00127-022-02308-9.

## Introduction

Due to the COVID-19 pandemic, countries worldwide adopted prevention strategies, most including physical distancing by limiting group sizes and closing facilities [1]. These lockdown strategies have had a major impact on social lives and mental health, several studies have already reported increased prevalence of loneliness, depression and anxiety in the general population [2–4]. Social and mental health are strongly connected [5–7], in patients with mental health problems social isolation has for example been associated with more severe symptoms. Additionally, a limited social capital, defined as the availability of social resources [8], was associated with a hampered recovery [5, 9]. We hypothesize that due to the lockdown multiple aspects of psychosocial health are affected and potentially also links between different psychosocial health constructs are rearranged.

Even though the interest in psychosocial health is growing, most studies have analyzed the impact of loneliness, social capital, and worry on mental health in separate models and as distinct psychosocial constructs [8]. Because of the overlap in symptoms, high co-occurrence and strong correlations between these constructs, growing evidence suggests it might be beneficial to study psychosocial health as a network [10], as these constructs might also need to be targeted in conjunction rather than separately. Using network analyses, the associations among psychosocial constructs are estimated in one network model, allowing the investigation of interrelationships among these constructs in the context of the full network of psychosocial health constructs. Crucially, in estimating an association between two constructs, association between the other constructs that are included in the network are taken into account, allowing the visualization of all associations at once and to distinguish direct from indirect effects [10, 11].

The structure of a psychosocial network might potentially not only be affected by the COVID-19 pandemic but also between persons of different age and sex, as lockdown measures may have impacted groups differently, for example because different advice for elderly persons [12, 13]. The impact of age on the effects of the lockdown and psychosocial health remains unclear, however [2, 14]. Results on the impact of sex on the risk of psychiatric symptoms during the lockdown have been inconsistent. Some studies report that women had an increased risk on anxiety and depression [2, 14], whereas others suggested men had an increased risk of depression [4].

This study aimed to identify the network of psychosocial health, by constructing a network of depressive symptoms, anxiety symptoms, loneliness, social connectedness, and pandemic-related worry in a large population-based sample of middle-aged and older adults. We added age and sex to assess differences in the network for these demographics.

## Methods

### Participants and design

We included participants from the Rotterdam study, an ongoing population-based cohort of middle-aged and older inhabitants of Rotterdam, the Netherlands. The study started in 1990 to investigate prevalence, history, and risk factors of common diseases later in life. So far, about 20,000 participants aged ≥ 40 years were included. Further details of the study design have been described elsewhere [15, 16]. The Rotterdam Study has been approved by the Medical Ethics Committee of the Erasmus MC (registration number MEC 02.1015) and by the Dutch Ministry of Health, Welfare and Sport (Population Screening Act WBO, license number 1071272-159521-PG). The Rotterdam Study has been entered into the Netherlands National Trial Register (NTR; www.trialregister.nl) and into the WHO International Clinical Trials Registry Platform (ICTRP; www.who.int/ictrp/network/primary/en/) under shared catalogue number NTR6831. All participants provided written informed consent to participate in the study and to have their information obtained from treating physicians.

During the COVID-19 pandemic, repeated questionnaires were sent to participants from April 20th, 2020 onwards [16], which included questions on psychosocial health. In the current project, we used data collected with the first questionnaire, which was sent out to 8732 participants of the Rotterdam Study. This number represents all participants who were alive by April 2020, excluding participants who lived in nursing homes. The response rate for the first questionnaire was 71.5%.

Of the 6241 participants that filled out the first questionnaire between of April, 22nd 2020 and July 16th, we excluded participants with more than one item missing for depressive symptoms or anxiety symptoms (*n* = 566), participants with missing items for loneliness, social connectedness and/or worry (*n* = 631), and participants who filled out the questionnaire after May 11th (*n* = 491) as restrictions were substantially eased at this date. This resulted in a total sample of 4553 eligible participants for analyses.

### Depressive symptoms

Depressive symptoms were assessed with the shortened version of the Center for Epidemiologic Studies Depression scale (CES-D), which consists of 10 items, rated on a 0–3 scale [17, 18]. The total score ranges from 0 to 30, with a higher score indicating more severe depressive symptoms. A weighted score was calculated if ≥ 9 of the questions were completed. If less than 9 of the questions were completed, CES-D was set to missing.

### Anxiety symptoms

Anxiety symptoms were assessed with a 7-item, rated on a 0–3 scale, version of the Hospital Anxiety and Depression Scale (HADS-A) questionnaire [19]. A weighted score was calculated if ≥ 6 of the questions were completed, if less than 6 of the questions were completed, HADS-A was set to missing.

### Loneliness

Loneliness was assessed with the three-item University of California, Los Angeles (UCLA) loneliness scale [20]. Additionally, the question “*How often do you feel alone*” was asked with the follow possible answers “*Almost never, or never*”, “*Sometimes*”, or “*Often*” (range 1–3). The questions were included as separate items in the network analyses.

### Social connectedness

Social connectedness was assessed an indicator for social capital, the availability of social resources [8]. In this study, social connectedness was assessed with five items. These items were assessed using the following statements (1) “*I feel connected to all Dutch people*”, (2) “*I feel connected to my neighbors, family or friends*”, (3) “*I receive the help and support that I need from my neighbors, family or friends*”, (4) “*I do everything I can to help others who are infected with the coronavirus*”, and (5) “*I expect others to do everything they can to help me if I become infected with the coronavirus*”. Each of these statements could be answered with “*Strongly disagree*”, “*Slightly disagree*”, “*Not agree, or disagree*”, “*Slightly agree*”, or “*Strongly agree*” (range 1–5). The questions were included as separate items in the network analyses.

### Pandemic-related worry

Pandemic-related worry was assessed with five items. These items were assessed with the following statements (1) “*I worry about contracting the virus myself*”, (2) “*I worry about someone close to me contracting the virus*”, (3) “*I am worried that myself or my relatives will encounter severe financial difficulties*”, (4) “*I am worried about the time it will take before it is possible to return to my daily routines*”, and (5) “*I am worried about not being able to visit my family or friends*”. Each of these statements could be answered with “*Never*”, “*Rarely*”, “*Sometimes*”, “*Often*”, or “*Almost Continuously*” (range 1–5).The questions were included as separate items in the network analyses.

### Other variables

Age and sex were self-reported within the questionnaire.

### Statistical analyses

Descriptive analyses are presented as number with percentage for categorical variables and mean with standard deviation (SD) for numerical data. To assess non-response, demographic and health characteristics of participants who were included in our analyses were compared to those of participants who were excluded because of missing data. Analyses were performed in R version R 3.6.3 (R Foundation for Statistical Computing, Vienna, Austria, www.R-project.org).

Networks were estimated using the MGM R package [21], and LASSO regularization was used to prevent inclusion of spurious edges [22]. Extended Bayesian Information Criterion (EBIC) was used to select the optimal tuning parameter, with the hyper-parameter set to 0.25. All variables included in a network were presented as a node. The estimated conditional direct associations were represented by edges, with a value between − 1 and 1. The thickness of the edges is scaled relative to the reference value of 0.1. This was the same for all networks, to be able to compare different networks. Conditional associations between each two variables were estimated taking into account all other variables in the network [10, 23]. These conditional associations can be used to estimate how different variables included in the network predict one another directly, but also indirectly via a third variable connecting these nodes [22, 24]. Therefore, in a network analyses, it can be explored how variables in a network are connected to one another, while taking into account all other variables in the network. By estimating what variables are most central, in other words most connected to other variables, it is possible to gain insight into what variables are most important in connecting clusters of variables. Furthermore, to estimate whether associations within the network significantly differed from one another, we performed an Edge-weight bootstrapped difference test [22].

The relevance of all edges connected to a node was assessed using a predictability estimation for each of the variables in our network [25]. For each node, the predictability was calculated as the proportion of variance in that variable that is explained by other variables in the network that are directly connected to it and is presented as a ring around the node representing that variable. The predictability was calculated for all variables in the network. A ring that is completely filled indicates 100% of the variance of a variable can be explained by the other variables in the network that are connected to it, whereas an empty ring indicates 0% of its variance can be explained by other variables in the network. We assessed estimated the stability of the estimated edges in the network, using the bootnet R package [22, 24] using nonparametric bootstrapping with 5000 bootstrap samples.

First, we estimated a network where the weighted depressive symptoms sum-score, weighted anxiety symptoms sum-score, and each of the described items for loneliness, social connectedness, and worry were included. This network provides insight into how depressive symptoms score and anxiety are conditionally associated to other items of psychosocial health during the lockdown. As the depressive symptoms score also includes an item on loneliness, we reran this network excluding the loneliness item from the depressive symptoms score. Given the high comorbidity between depressive symptoms and anxiety symptoms [11], it is to be expected that these constructs will be strongly associated in the network, and explain much of the variance in one another. To estimate the amount in which items of loneliness, social connectedness or worry explain the variance of depressive symptoms score and anxiety symptoms score, we estimated separate networks including either depressive symptoms score or anxiety symptoms score.

Second, we aimed to identify if age and sex affected the network structure as described above. To do this, we estimated a network tree using the networktree R package [26]. Using this method, the sample is split recursively based on pre-specified binary variables (i.e., age and sex in this study). The networktree function estimates whether the network structure is significantly affected by either of the specified variables. The function estimates in what order the study population should be split based on these variables to maximize the differences between the network structures. For each split, the significance of the difference between the groups of that variable was presented as a p value. Differences between groups were assessed with visual inspection and by comparing the edge-weights of the networks. Significance of the observed differences of individual edges between the different networks was statistically tested using the Network Comparison Test [27]. In this study, we used sex and age group as potential covariates, where for age group, participants were categorized as either (1) the median age of 69 or below, or (2) above the median age of 69 years.

Third, the scores of depressive symptoms consist of a heterogeneous spectrum of symptoms. Therefore, we aimed to gain insight into what depression and anxiety symptoms were most strongly associated to items of loneliness, social connectedness or worry. Furthermore, this network allows us to identify what symptoms of anxiety or depression connect these constructs to items of loneliness, social connectedness or worry, and the cluster of depression or anxiety symptoms. We estimated a network including all loneliness items, all social connectedness items, all pandemic-related worry items, all depressive symptoms, and all anxiety symptoms separately.

For all Figures, except Supplementary Fig. 5, the coloring of variables in the network was presented based on the categorization in the questionnaire. Clustering of variables was further explored using Walktrap and Spinglass algorithms, which are the most commonly used algorithms for community detection in networks [28]. The Walktrap algorithm is a hierarchical algorithm that identifies communities in the network via random walks based on the distance between nodes [28, 29]. The Spinglass algorithm is an optimization method that relies on resemblance between statistical mechanics of the network [29]. For networks with a small number of nodes, the Walktrap and Spinglass algorithms are considered most accurate [28]. We used both algorithms to estimate robustness of the clusters that were observed.

## Results

In this study, we included 4553 participants with a mean age of 68.6 ± 11.2 years and 56% were women (Table 1). Participants included in the analyses were on average younger (68.6 ± 11.2 vs 72.7 ± 12.2 years, *t* = 11.95, *p* < 0.001), more often men (43.9 vs 35.6%, *χ*^2^ = 34.21, *p* < 0.001), and reported less depressive symptoms (5.2 ± 4.6 vs 5.8 ± 5.0, *t* = − 2.51, *p* = 0.012) compared to participants with incomplete data. There were no significant differences in terms of employment, physical health status, and anxiety symptoms. Participants included in our analyses reported a median depressive symptoms score of 4 (IQR = 2–8) and a median anxiety symptoms score of 3 (IQR = 1–5). Furthermore, 743 (16.3%) exceeded the cut-off score of 10 for clinically relevant depressive symptoms and 714 (15.7%) exceeded the cut-off score of 7 for clinically relevant anxiety symptoms. In total, 483 (10.6%) participants exceeded the clinical cut-off for both depressive symptoms and anxiety symptoms score.

**Table 1:** Demographics of the study population (*N* = 4553)

	*N*	(%)	Median	IQR
Age (years)^a^			68.6	11.2
Women	2,556	56.2		
Psychopathology				
Depressive symptoms (score)^b^			4	2–8
Anxiety symptoms (score)^c^			3	1–5
Loneliness^d^				
Feeling left out			1	1–2
Feeling isolated			2	1–2
Feeling alone			1	1–2
Missing company			2	1–2
Social connectedness^e^				
Connected to all Dutch people			4	3–5
Connected to neighbors, friends or family			5	4–5
Currently receiving help and support from family or friends			4	3–5
Currently offering help to others			3	2–4
Expecting help from others if needed in case of a COVID-19 infection			4	3–5
Pandemic-related worry^f^				
Worry to get infected with COVID-19			3	2–3
Worry others getting infected with COVID-19			3	2–3
Financial worry			2	1–3
Worry about daily life			3	2–3
Worry about inability to visit family or friends			3	2–4

*IQR* inter quartile range, *SD* standard deviation

^a^Mean and SD

^b^Assessed using the 10-item Center for Epidemiologic Studies Depression scale

^c^Assessed using the Hospital and Depression Scale

^d^Possible answering options were “Almost never, or never” = 1, “Sometimes” = 2, and “Often” = 3

^e^Possible answering options were “Strongly disagree” = 1, “Slightly agree” = 2, “Neutral” = 3, “Slightly agree” = 4, and “Strongly agree” = 5

^f^ Possible answering options were “Never” = 1, “Rarely” = 2, “Sometimes” = 3, “Often” = 4, and “Almost continuously” = 5

**Fig. 1:**
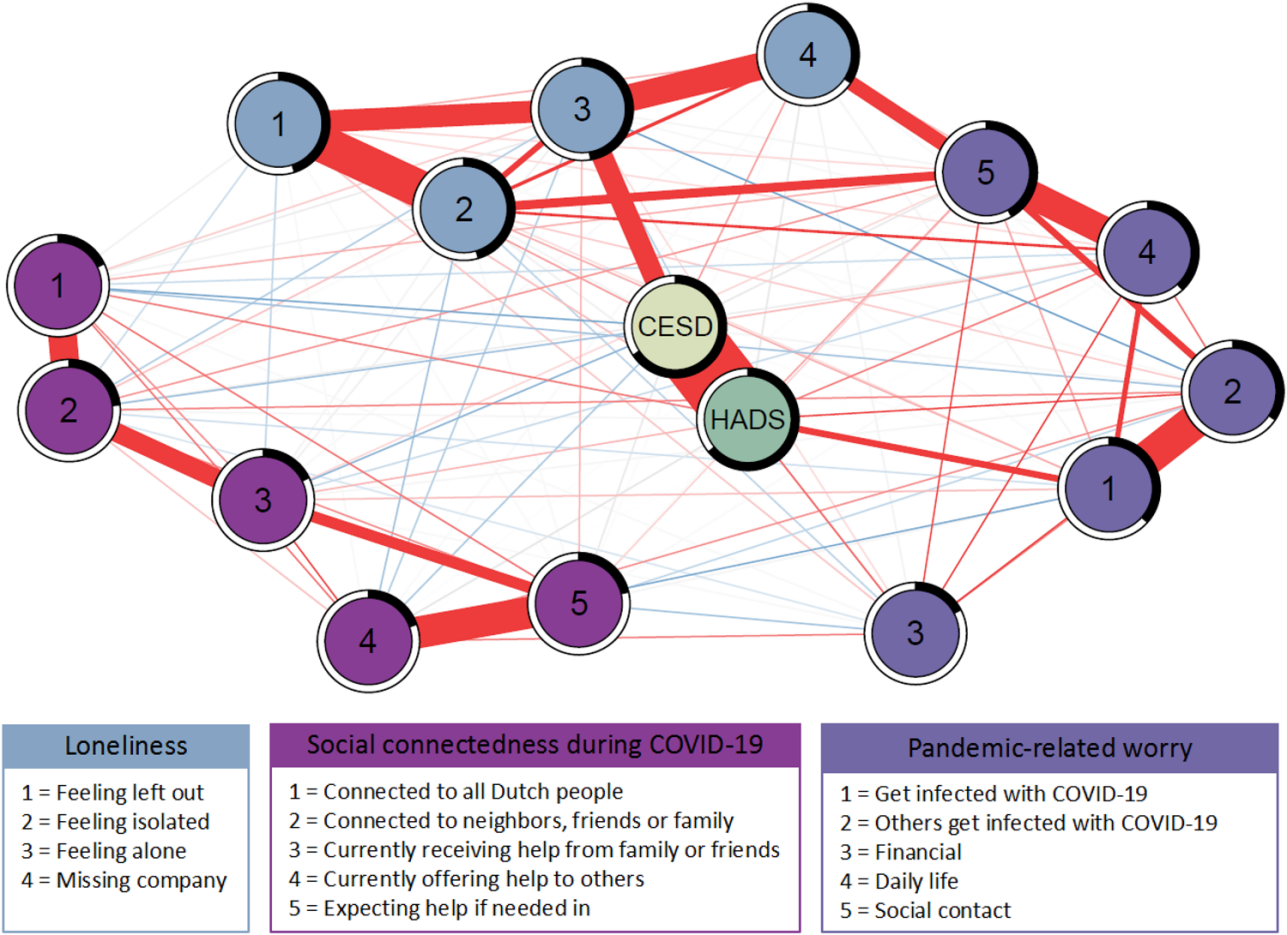
Network of psychosocial health factors during the first COVID-19 lockdown. The estimated network of depressive symptoms score, anxiety symptoms score, and items of loneliness, social connectedness, and worry. Each variable is represented by a node in the network. Direct, conditional positive associations (red) and negative associations (blue) between variables are indicated with edges. Strength of the association is indicated by thickness of the edge, using a correlation value of 0.1 as maximum value reference point. For each node the predictability, indicating the proportion of variability that is explained by other variables in the network it is connected to, is presented as a ring around the node. A completely filled ring (100%) indicates all variance of a variable can be explained by the other variables in the network, whereas an empty ring (0%) indicates none of the variance is explained. Explained variance (%) per variable: depressive symptoms score (CES-D), 66%; anxiety symptoms score (HADS), 64%; feeling left out, 45%; feeling isolated, 45%; feeling alone, 47%; missing company, 34%; connected to all Dutch people, 18%; connected to neighbors, friends, and family, 23%; receiving help, 18%; offering help, 20%; expecting help, 21%; worry to get infected, 37%; worry others get infected, 34%; financial worry, 17%; worry about daily life, 38%; worry about social contact, 42*%*

First, we estimated the overall network including the depressive symptoms score, anxiety symptoms score, and separate items for loneliness, social connectedness, and pandemic-related worry (Fig. 1). After exclusion of the loneliness item in the depressive symptoms score, the network remained unchanged (data not shown). Within the overall network, we observed depressive symptoms score and anxiety symptoms score were central in the network, surrounded by clusters for items of loneliness, items of social connectedness, and items of pandemic-related worry. These clusters were confirmed by the Walktrap and Spinglass clustering algorithms (data not shown). We observed that depressive symptoms score was positively associated with the loneliness items ‘*feeling left out*’ (edge-weigh* t* = 0.05), ‘*feeling alone*’ (edge-weight = 0.25), and ‘*missing company*’ (edge-weight = 0.05). Additionally, depressive symptoms score was negatively associated with the social connectedness items ‘*feeling connected to all Dutch people*’ (edge-weight = -0.07*)*, ‘*feeling connected to neighbors, friends or family*’ (edge-weight = − 0.05), ‘*receiving help*’ (edge-weight = − 0.06), and ‘*offering help*’ (edge-weight = − 0.04) (Fig. 1, Supplementary Table 1). Anxiety symptoms score was positively associated with all the pandemic-related worry items: ‘*worry to get infected with COVID-19*’ (edge-weight = 0.14), ‘*worry others will get infected with COVID-19*’ (edge-weight = 0.10), ‘*financial worry*’ (edge-weight = 0.08), ‘*worry about daily life*’ (edge-weight = 0.07), and ‘*worry about social life*’ (edge-weight = 0.05). We observed the loneliness items ‘*feeling isolated*’ (edge-weight = 0.16), and ‘*missing company*’ (edge-weight = 0.20) were positively associated with the pandemic-related worry item ‘*worry about social contact*’ (Fig. 1, Supplementary Table 1). The edge-weights for all the edges in the network can be found in Supplementary Table 1. Depressive symptoms score was explained for 66% by variables in the network that were directly connected to it, for anxiety this was 64%. However, it is likely these explained variances are with a large proportion accounted by the strong relation between depressive symptoms score and anxiety symptoms score (edge-weight = 0.67). Using the Edge-weight bootstrapped difference test, we observed that the edge between depressive symptoms score and the anxiety symptoms score was significantly stronger than for all other edges within the network (Supplementary Fig. 1). To account for this, we estimated separate networks including either depressive symptoms score or anxiety symptoms score. In these separate networks, 37% of the variation in the depressive symptoms score could be accounted for by variables in the network that were directly connected to it (Supplementary Fig. 2). When depressive symptoms score was not included in the network, explained variance for anxiety symptoms score was 35% (Supplementary Fig. 3). Explained variance for the loneliness items was on average 43%, for social connectedness items this was 20% and for pandemic-related worries 33%. The nonparametric bootstrap showed that the bootstrapped sampling distributions around the estimated edges in our network were generally small, indicating relatively stable estimates (Supplementary Fig. 4).

**Fig. 2:**
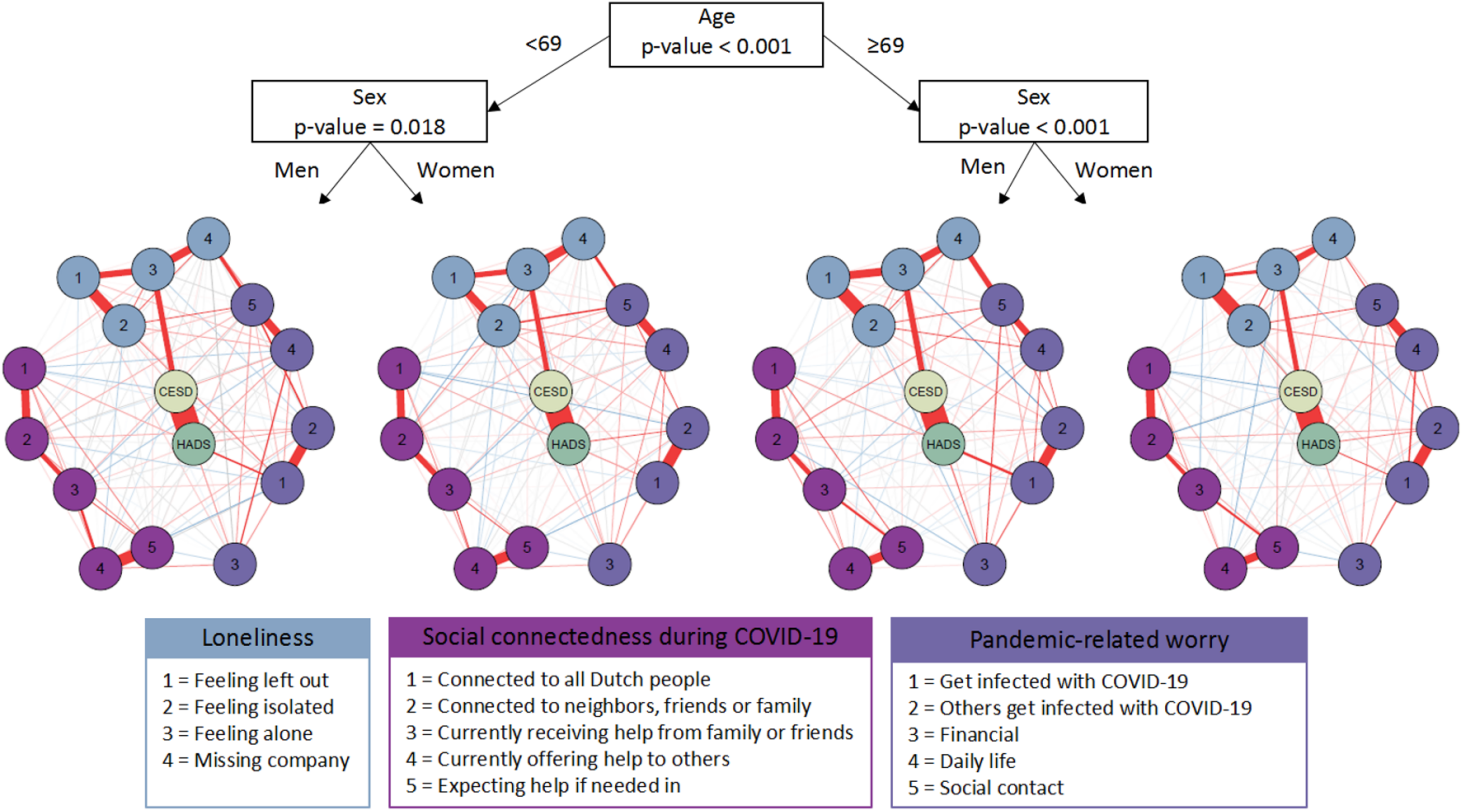
Networktree of psychosocial health factors during the first COVID-19 lockdown, based on differences between age and sex groups. The estimated network tree, using age and sex as potential covariates. Each network contains depressive symptoms score, anxiety symptoms score, and items of loneliness, social connectedness, and worry, represented by a node in the network. Direct, conditional positive associations (red) and negative associations (blue) between variables are indicated with edges. Strength of the association is indicated by thickness of the edge, using a correlation value of 0.1 as maximum value reference point

Second, we assessed whether the network structure was significantly different for people of different age or sex by estimating a networktree. The observed network structure significantly differed between age and sex (Fig. 2). To maximize the differences between the network structures of the groups, the population was first split based on age using the networktree function. The overall network for participants < 69 years significantly differed from the overall network of participants ≥ 69 years (*p* < 0.001). In both age groups, there was a second split based on sex. We observed that the overall network significantly differed between men and women in participants < 69 years (*p* = 0.018), and in participants ≥ 69 years (*p* < 0.001) (Fig. 2). In all networks, we observed an association of a higher depressive symptoms score with more ‘*feeling alone*’, less ‘*feeling connected to all Dutch people*, and less ‘*receiving help from others*’; and of a higher anxiety symptoms score with more ‘*worry to get infected with COVID-19*’, more ‘*financial worry*’, more ‘*worry about daily life*’ and more ‘*worry about social life*’ (Fig. 2, Supplementary Table 1). When comparing participants of < 69 years with those of ≥ 69 years, we observed that the association of depressive symptoms score with ‘missing company’ was stronger in participants of ≥ 69 years (difference in edge-weight = 0.06, *p* value = 0.001), whereas the associations of depressive symptoms with ‘feeling isolated (difference in edge-weight = 0.02, *p* value = 0.006), ‘financial worry’ (difference in edge-weight = 0.06, *p* value = 0.022) and ‘worry about daily life’ (difference in edge-weight = 0.04, *p* value = 0.05) were stronger in participants < 69 years. Furthermore, we observed that the associations of depressive symptoms with ‘feeling connected to neighbors, family or friends’(difference in edge-weight = 0.04, *p* value = 0.10) and ‘worry to get infected with COVID-19’ (difference in edge-weight = 0.02, *p* value = 0.03) were stronger in women ≥ 69 years when compared to all other groups, whereas the association of depressive symptoms with ‘receiving help from others’ was significantly weaker (difference in edge-weight = 0.04, *p* value = 0.03). The association between high anxiety symptoms score and ‘worry to get infected with COVID-19’ was significantly weaker in women < 69 years opposed to all other groups (difference in edge-weight = 0.08, *p* value = 0.001), whereas the association between anxiety symptoms score and ‘worry others get infected with COVID-19’ was significantly stronger in women than in men (difference in edge-weight = 0.06, *p* value = 0.014). Furthermore, we observed that the association between anxiety symptoms score and ‘financial worry’ was substantially stronger in men than in women (difference in edge-weight = 0.04, *p* value = 0.10) (Fig. 2, Supplementary Table 1).

**Fig. 3:**
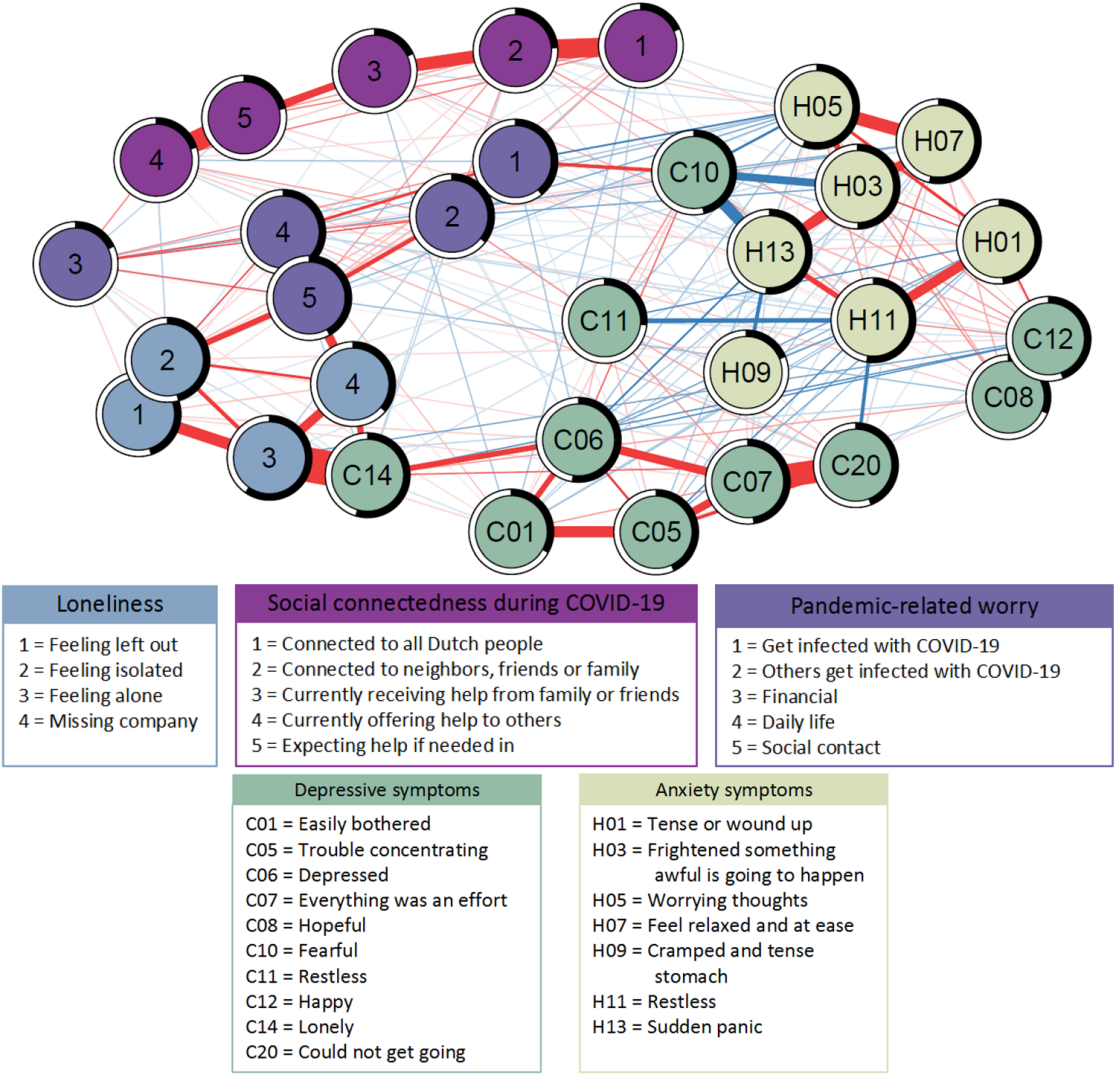
Network of psychosocial health factors during the first COVID-19 lockdown, including symptoms of depression and anxiety separately. The estimated network of depressive symptoms, anxiety symptoms, items of loneliness, items of social connectedness, and items of pandemic-related worry. Each variable is represented by a node in the network. Direct, conditional positive associations (red) and negative associations (blue) between variables are indicated with edges. Strength of the association is indicated by thickness of the edge, using a correlation value of 0.1 as maximum value reference point. For each node the predictability, indicating the proportion of variability that is explained by other variables in the network it is connected to, is presented as a ring around the node. A completely filled ring (100%) indicates all variance of a variable can be explained by the other variables in the network, whereas an empty ring (0%) indicates none of the variance is explained. Explained variance (%) per variable: C01, 33%; C05, 43%; C06, 52%; C07, 48%; C08, 31%;C10, 46%; C11, 27%; C12, 44%;C14, 55%; C20, 44%; H01, 48%; H03, 50%; H05, 55%; H07, 54%; H09, 19%; H11, 53%; H13, 51%; feeling left out, 45%; feeling isolated, 46%; feeling alone, 60%; missing company, 36%; connected to all Dutch people, 18%; connected to neighbors, friends, and family, 24%; receiving help, 18%; offering help, 20%; expecting help, 21%; worry to get infected, 39%; worry others get infected, 36%; financial worry, 18%; worry about daily life, 39%; worry about social contact, 42%

Last, we estimated a network including all depressive symptoms and anxiety symptoms separately, and all items of loneliness, items of social connectedness and pandemic-related worry as nodes, to assess which specific depressive symptoms and anxiety symptoms most strongly connect to other aspects of psychosocial health (Fig. 3). Based on the Walktrap clustering algorithm, we observed several clusters of variables; one with items of social connectedness, one with items of pandemic-related worry, and one with items of loneliness including the depressive symptom ‘*feeling lonely*’. Additionally, depressive symptoms and anxiety symptoms were divided over three clusters (Supplementary Fig. 5). We furthermore observed that the depressive symptoms that were reflecting a lack of positive affect (‘*feeling happy*’) and loneliness (‘*feeling lonely*’) connected depressive symptoms to loneliness items, whereas the depressive symptoms ‘*being easily bothered*, and ‘*feeling restless*, connected depressive symptoms to social connectedness (Fig. 3). Additionally, we observed that the depressive symptoms ‘*feeling fearful*’ and ‘*feeling restless*’, and the anxiety symptoms ‘*feeling frightened something awful is going to happen*, ‘*feeling relaxed and at ease*’, and ‘*having worrying thoughts*’ connected anxiety to pandemic-related worry (Fig. 3).

## Discussion

We demonstrated that within a network including multiple aspects of psychosocial health, a higher depressive symptoms score was specifically associated with items of loneliness and social connectedness, whereas a higher anxiety symptoms score was specifically associated with items of pandemic-related worry. Furthermore, items of loneliness were associated with items of social connectedness and items of pandemic-related worry. Second, the network structure significantly differed between age groups and sex, with for example a stronger association between anxiety symptoms score and ‘*worry to get infected*’ and ‘*financial worry*’ in men and an association between depressive symptoms score and ‘*worry about daily life*’ only in middle-aged participants. Last, when including depressive symptoms and anxiety symptoms in the network as individual items, we observed mainly depressive symptoms to connect the items of different psychosocial health constructs.

Depressive symptoms and anxiety are highly prevalent in the general population [30], and even more often reported during the COVID-19 lockdown [14]. Our results demonstrate that when taking all variables into account simultaneously, depressive symptoms score is particularly associated with loneliness and social connectedness, while anxiety is particularly associated with pandemic-related worry. These findings are partly in line with previous literature reporting depression and anxiety both to be associated with loneliness, social connectedness, and worry [8, 9, 31]. An explanation for our findings could be an overlap between constructs that we observed to be associated. Loneliness, social connectedness, and depression are constructs more based on self-worth and avoidance [32, 33]. People with more avoidant attachment styles or higher levels of self-disgust might be more likely to feel less socially connected, become more isolated during a lockdown, and at the same time develop a somber mood [32, 33]. Worry and anxiety both are constructs that are based on hyperarousal [34]. People with more neurotic personality traits and vulnerability to hyperarousal might be more likely to worry about the COVID-19 pandemic more, and at the same time feel more anxious about it [34, 35]. However, because of the strong association between depression and anxiety, comorbid symptoms are likely to occur over time [36, 37].

We observed age and sex significantly altered the network structure. First, in older men and women, the network structures were less dense when compared to middle-aged men and women. A denser network indicates that change in either variable has a stronger influence on the network structure, than in a less dense network. An explanation for more dense networks for middle-aged men and women could be a bigger impact of the lockdown on their daily life. Middle-aged people are more likely to have a job and, therefore, experience a substantial change in their daily routines and stronger job insecurity, whereas the older people in our sample were frequently retired. This was also supported by the associations of depressive symptoms with worry about social contact and daily life in participants < 69 years, which were absent in participants ≥ 69 years. Second, based on our findings, it seems depressive symptoms and anxiety are more strongly associated with the social aspects, such as strong social connectedness, in women. Whereas in men, practical aspects like financial security seem more important. These implications are supported by gender-based personality traits [38]. Compared to men, women tend to report a lower self-esteem and the experience of more distressing emotions and thus might be more vulnerable to mood disorders and loneliness, especially in response to stressful events such as a pandemic [38, 39]. Additionally, women tend to be more focused to care for others, potentially putting them at a higher risk to experience social distress, whereas men might have a stronger focus on financial concerns [38]. However, it might well be that the observed differences can also be in part explained by differences in the extent to which men and women feel at ease to report these symptoms. Third, a higher anxiety symptoms score was associated with worry to get infected with COVID-19 in all groups, but this association was substantially weaker in women < 69 years opposed to all other groups. Furthermore, we observed anxiety was associated with worry others will get infected with COVID-19 in all groups, except older men. An explanation could be that risks for severe consequences of a COVID-19 infection were reported to be specifically high in middle-aged men and older adults, causing them to feel more anxious about possible consequences and worry more about themselves and feel more anxious about possible consequences [12, 13].

When we included symptoms of depression and anxiety as separate items in the network, we observed multiple clusters of items. For most psychosocial health constructs, we observed that items within a construct indeed clustered together. Items for depression however were more spread throughout the network, suggesting that depressive symptom items connect the different constructs. Potentially, this is because in this network, depressive symptoms are assessed with a substantially larger number of items than the other constructs, therefore, allowing more variance. Another explanation could be that depressive symptoms cover a broader spectrum of psychosocial health complaints, because items can be classified as related to either (1) feeling depressed and lonely, (2) somatic, (3) interpersonal and connected to others, (4) positive affect and self-worth, or (5) feeling fearful [18, 40].

Together, these findings seem to suggest that depressive symptoms play a central role in psychosocial health. Therefore, detection, prevention and treatment efforts might want to focus on of depressive symptoms, as these tie in to many other complaints. However, any approach should be tailored to the specific needs of the person. Moreover, these findings also emphasize that psychosocial factors should be considered in conjunction rather than as stand-alone complaints. Future research into prevention and treatment efforts should take these notions into account.

Several limitations need to be taken into account when interpreting these results. First, items of loneliness, social connectedness, and pandemic-related worry were only assessed during the COVID-19 pandemic. Therefore, we were not able to compare the network to a network prior to the lockdown. Second, questions on mental health were frequently missing due to non-response. Therefore, there is a possibility of selection bias as the choice to leave mental health questions unanswered is unlikely to be completely random. Lastly, exclusion of participants who responded after May 11th potentially introduced selection bias, as participants who take longer time to respond might differ from those responding earlier. Additionally, we observed that participants included in our analyses were younger, more often, men and reported less depressive symptoms compared to participants that were excluded because of incomplete data. Therefore, our results might not be representative for the entire Rotterdam study population. Nevertheless, performing a network analysis including multiple aspects of psychosocial health using a large population-based cohort of middle-aged and older adults is unique in this field and allowed us to assess underlying associations.

Altogether, in our study population of middle-aged and older adults, we demonstrated that within a network of psychosocial factors, a high depressive symptoms score was most strongly associated with loneliness and poorer social connectedness, whereas a high anxiety symptoms score was most strongly associated with pandemic-related worry. This emphasizes that psychosocial factors should be considered in conjunction for both population-based mental health prevention efforts and treatment efforts in clinical care. Importantly, we showed that the lockdown had a stronger impact on middle-aged participants compared to older participants, women were more vulnerable to social distress, and men were more vulnerable to distress about practical issues. Efforts for prevention and treatment might, therefore, need to be tailored to specific demographic groups.

## Supplementary Information

Below is the link to the electronic supplementary material.

Supplementary file1 (DOCX 1623 KB)
