# Supplementary material for: The network of psychosocial health in middle-aged and older adults during the first COVID-19 lockdown
Source: Soc Psychiatry Psychiatr Epidemiol. 2022 Jun 8;57(12):2469–79. doi: 10.1007/s00127-022-02308-9 (PMC9174915; doi:10.1007/s00127-022-02308-9)
Supplement: Supplementary file 1 — Supplementary file1 (DOCX 1623 KB) [file 127_2022_2308_MOESM1_ESM.docx]

# Supplement


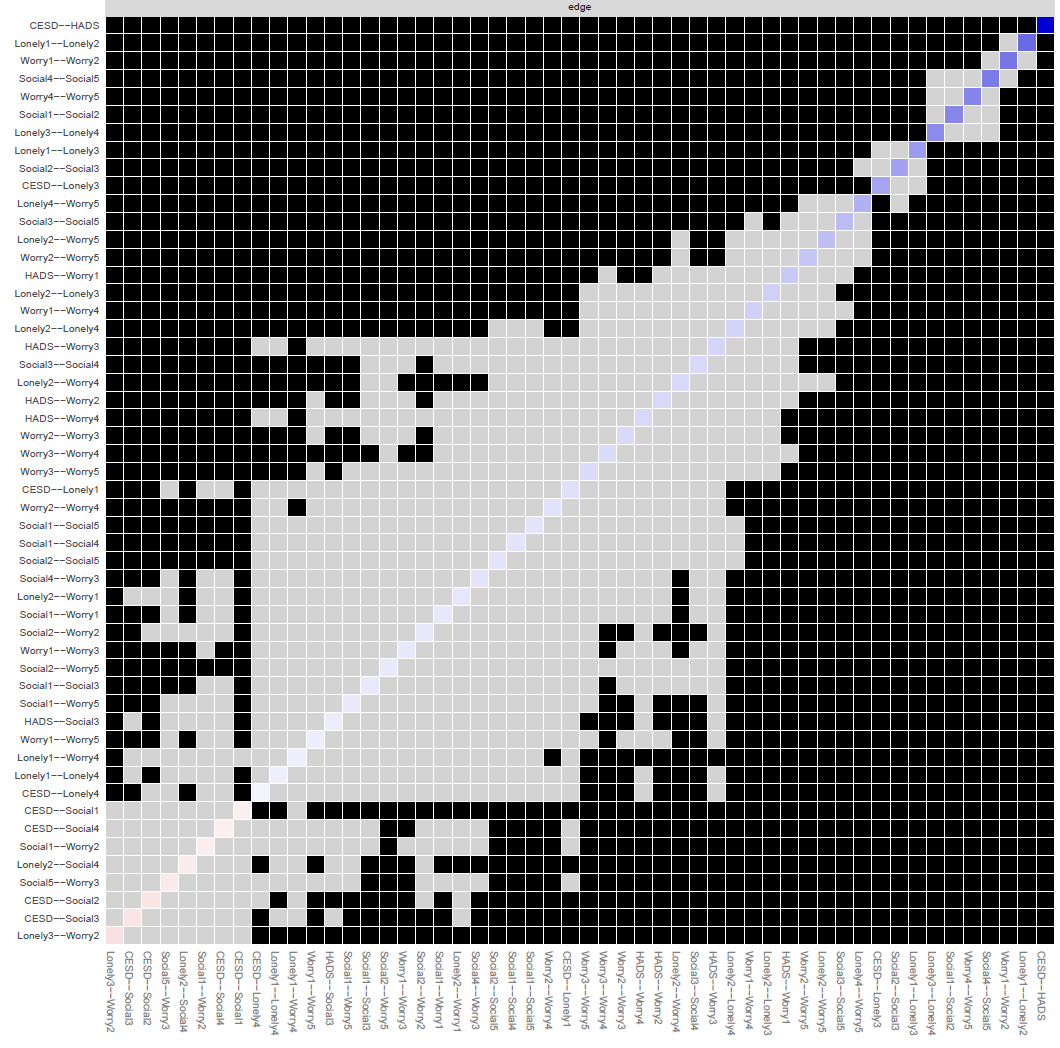


**Supplementary Figure 1**:**Results of the edge-weight bootstrapped difference test**

Results of the bootstrapped difference tests (α = 0.05) for all edge-weights that were non-zero in the estimated network. Edges that differ significantly from each other are indicated by a black box, whereas edges that do not differ significantly from each other are indicated by a gray box. The colored boxes in the middle of the figure indicated whether the conditional associations between the named variables were positive (blue) or negative associations (red), where a brighter color indicates a stronger association.


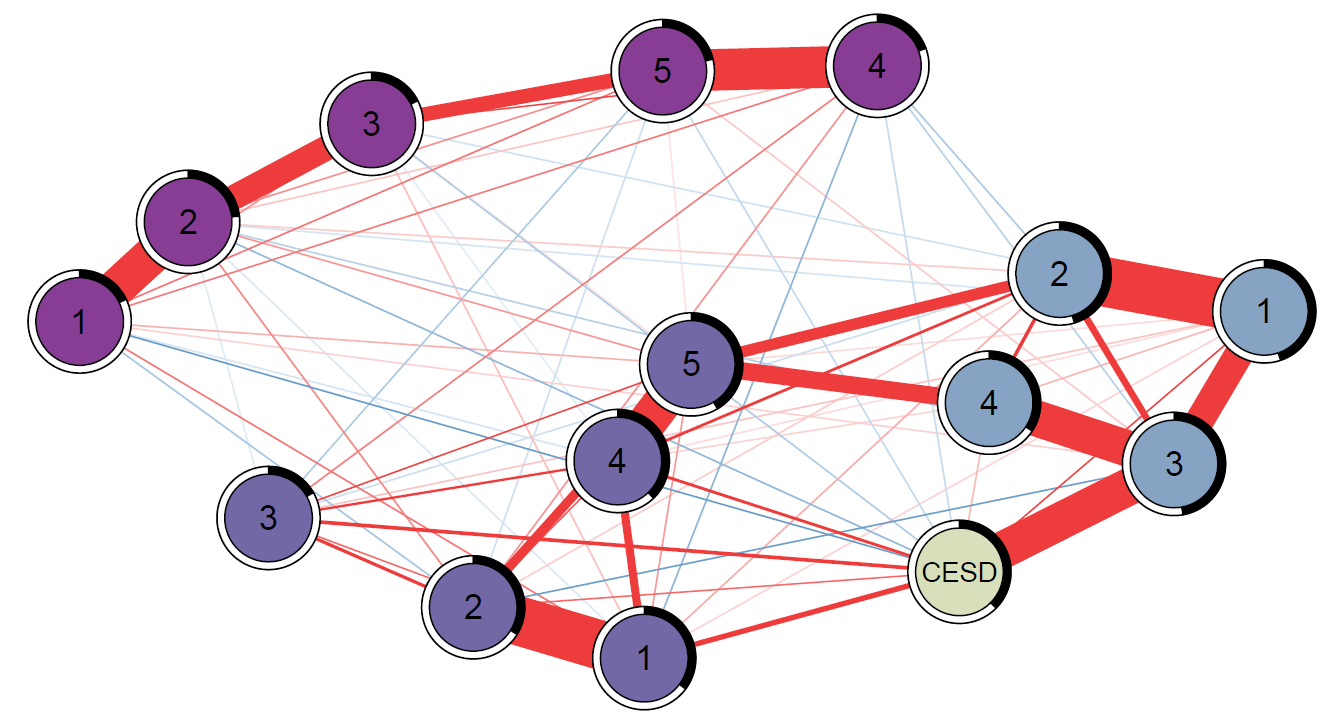


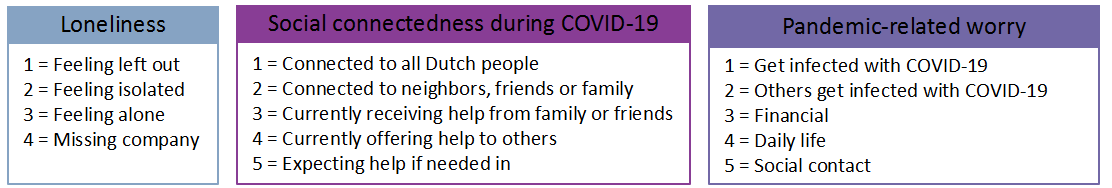


**Supplementary Figure 2**: **Network of psychosocial health factors and depressive symptoms score**

The estimated network of depressive symptoms score and items of loneliness, social connectedness, and worry. Each variable is represented by a node in the network. Direct, conditional positive associations (red) and negative associations (blue) between variables are indicated with edges. Strength of the association is indicated by thickness of the edge, using a correlation value of 0.1 as maximum value reference point. For each node the predictability, indicating the proportion of variability that is explained by other variables in the network it is connected to, is presented as a ring around the node. A completely filled ring (100%) indicates all variance of a variable can be explained by the other variables in the network, whereas an empty ring (0%) indicates none of the variance is explained. Explained variance (%) per variable: depressive symptoms score (CESD), 37%; feeling left out, 45%; feeling isolated, 45%; feeling alone, 47%; missing company, 34%; connected to all Dutch people, 18%; connected to neighbors, friends, and family, 23%; receiving help, 18%; offering help, 20%; expecting help, 21%; worry to get infected, 35%; worry others get infected, 34%; financial worry, 17%; worry about daily life , 38%; worry about social contact, 42%.


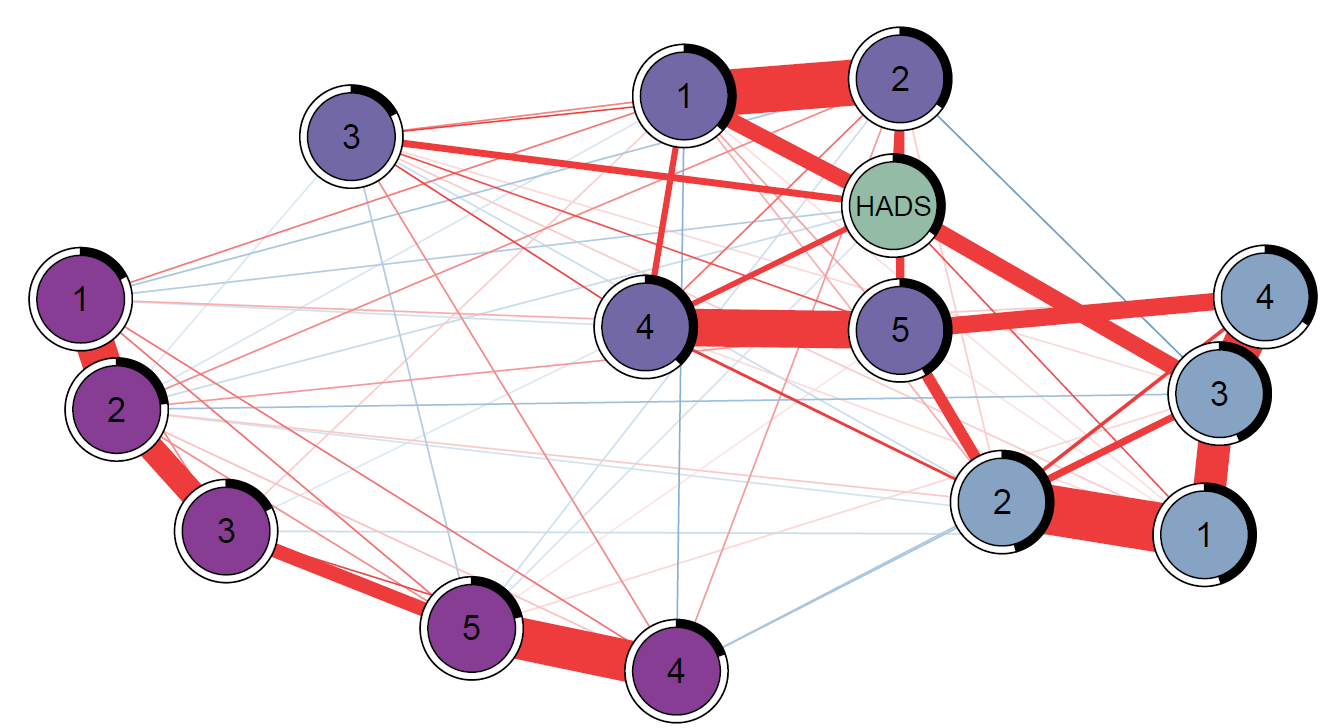


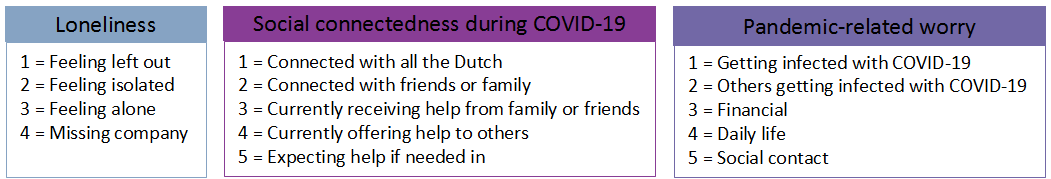


**Supplementary Figure 3**: **Network of psychosocial health factors and anxiety symptoms score**

The estimated network of anxiety symptoms score and items of loneliness, social connectedness, and worry. Each variable is represented by a node in the network. Direct, conditional positive associations (red) and negative associations (blue) between variables are indicated with edges. Strength of the association is indicated by thickness of the edge, using a correlation value of 0.1 as maximum value reference point. For each node the predictability, indicating the proportion of variability that is explained by other variables in the network it is connected to, is presented as a ring around the node. A completely filled ring (100%) indicates all variance of a variable can be explained by the other variables in the network, whereas an empty ring (0%) indicates none of the variance is explained. Explained variance (%) per variable: anxiety symptoms score (HADS), 35%; feeling left out, 45%; feeling isolated, 46%; feeling alone, 44%; missing company, 34%; connected to all Dutch people, 18%; connected to neighbors, friends, and family, 23%; receiving help, 17%; offering help, 20%; expecting help, 21%; worry to get infected, 37%; worry others get infected, 35%; financial worry, 17%; worry about daily life , 38%; worry about social contact, 42%.


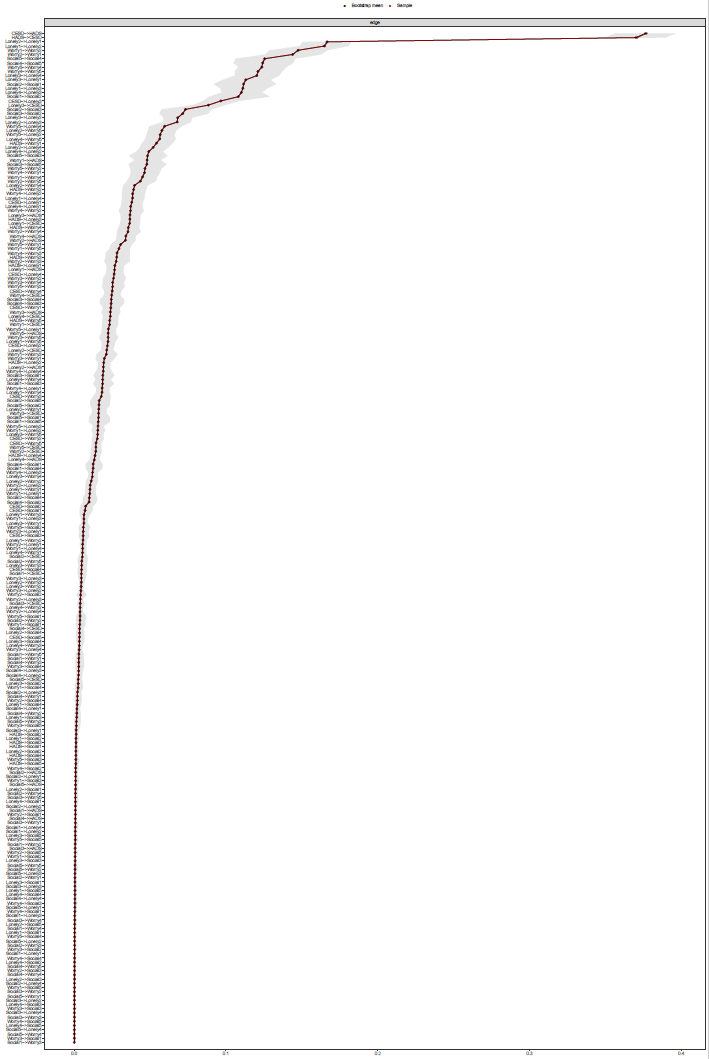


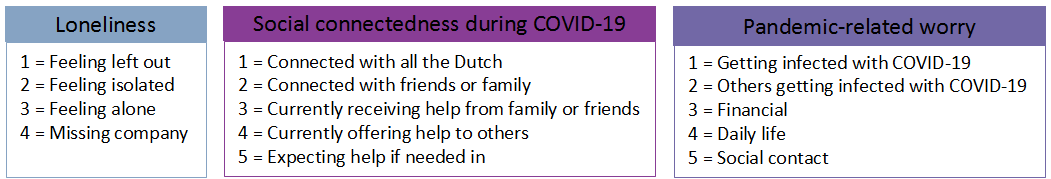


**Supplementary Figure 4**: **Confidence Intervals estimated using 5,000 bootstrap samples**

The bootstrapped confidence intervals of the estimated edge-weights in our network of depressive symptoms score, anxiety symptoms score and items of loneliness, social connectedness, and worry. The red line indicates the sample values, the black line the bootstrapped mean, and the gray area the bootstrapped CIs. The edges are ordered from the edge with the highest weight to the edge with the lowest weight.


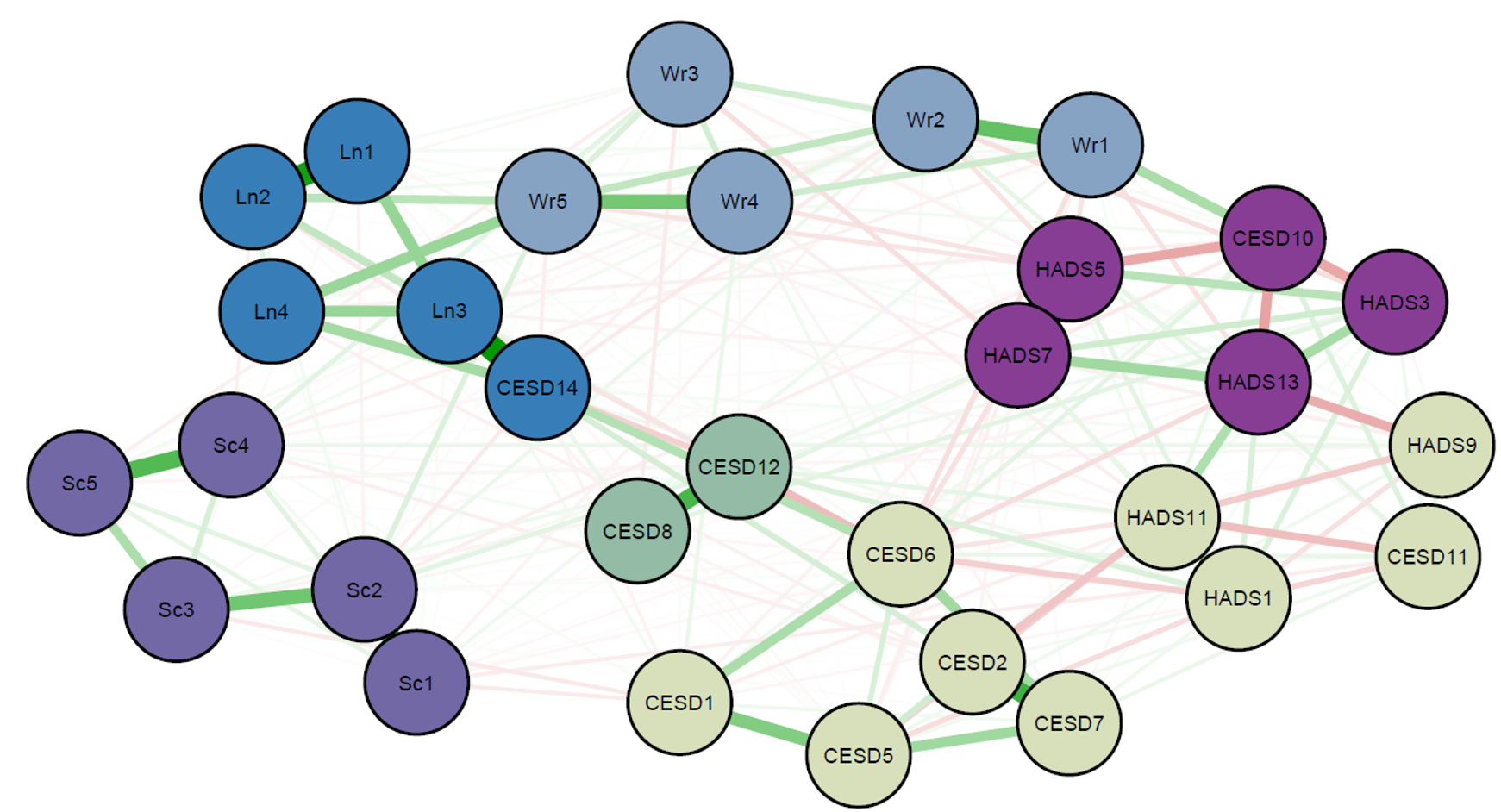


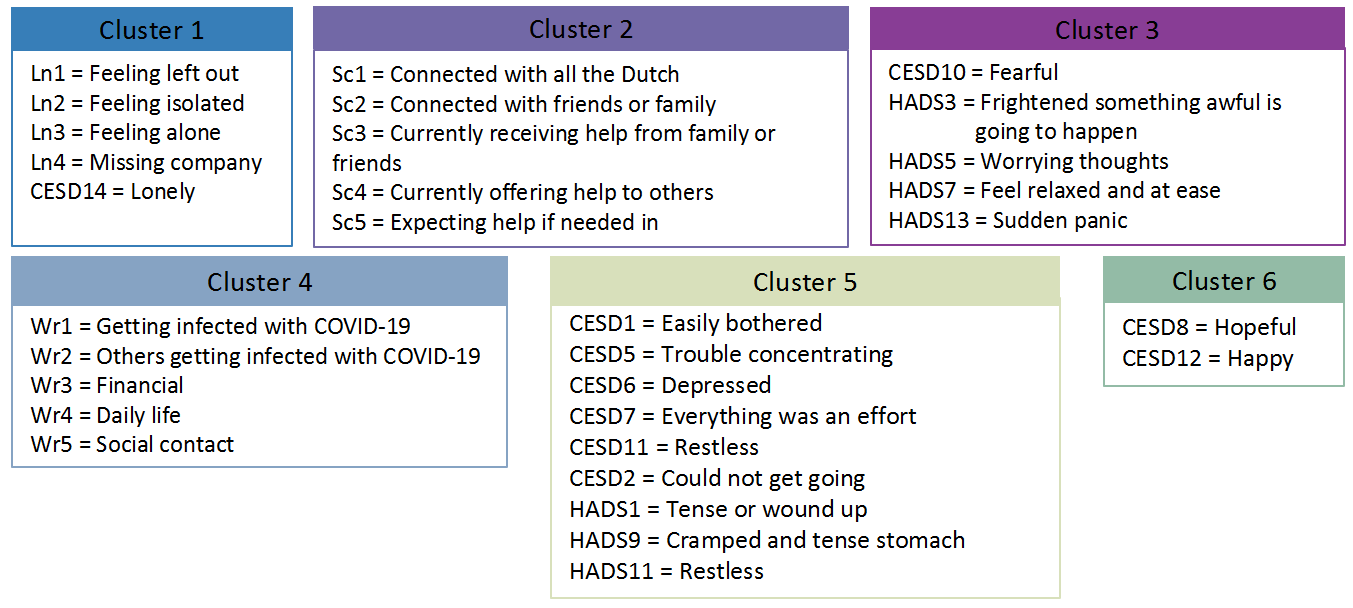


**Supplementary Figure 5**: **Clustering of items according to the Walktrap clustering algorithm**

The estimated network of depressive symptoms score, anxiety symptoms score, and items of loneliness, social connectedness, and worry. Items with the same color cluster together, as estimated using the EGA Walktrap clustering algorithm.

**Supplementary Table 1.** Overview of the edge-weights for different networks. Item number is presented in parentheses with each item.

| **From** | **To** | **Total sample**  **(N=4,553)** | **Men**  **<65**  **(N=980)** | **Women <65**  **(N=1,312)** | **Men**  **≥69**  **(N=1,017)** | **Women**  **≥69**  **(N=1,244)** |
| --- | --- | --- | --- | --- | --- | --- |
| CESD | HADS | 0,67 | 0,66 | 0,70 | 0,63 | 0,65 |
| CESD | Left out (Lonely1) | 0,05 | 0,04 | 0,01 | 0,03 | 0,11 |
| CESD | Isolation (Lonely2) | -0,01 | 0,03 | 0,02 | -0,02 | -0,08 |
| CESD | Alone (Lonely3) | 0,25 | 0,26 | 0,25 | 0,26 | 0,24 |
| CESD | Miss company (Lonely4) | 0,05 | 0,02 | 0,03 | 0,06 | 0,07 |
| CESD | Connect Dutch (Social1) | -0,07 | -0,07 | -0,06 | -0,03 | -0,10 |
| CESD | Connect friends (Social2) | -0,05 | -0,03 | -0,02 | -0,04 | -0,11 |
| CESD | Receive help (Social3) | -0,06 | -0,06 | -0,09 | -0,06 | -0,02 |
| CESD | Help others (Social4) | -0,04 | -0,03 | -0,04 | -0,03 | -0,06 |
| CESD | Expect help (Social5) | -0,02 | 0,00 | 0,01 | -0,03 | -0,06 |
| CESD | Infection (Worry1) | 0,00 | -0,05 | 0,01 | -0,03 | 0,04 |
| CESD | Others infected (Worry2) | -0,01 | -0,02 | -0,03 | 0,04 | -0,02 |
| CESD | Finance (Worry3) | 0,03 | 0,06 | 0,07 | -0,06 | 0,02 |
| CESD | Daily life (Worry4) | 0,04 | 0,07 | 0,05 | 0,00 | 0,01 |
| CESD | Social life (Worry5) | -0,02 | -0,07 | -0,03 | 0,01 | -0,01 |
| HADS | Left out (Lonely1) | 0,03 | 0,01 | 0,08 | 0,02 | 0,01 |
| HADS | Isolation (Lonely2) | 0,02 | 0,00 | 0,00 | 0,02 | 0,04 |
| HADS | Alone (Lonely3) | -0,03 | -0,02 | -0,06 | -0,06 | -0,01 |
| HADS | Miss company (Lonely4) | -0,03 | -0,02 | -0,05 | 0,00 | -0,01 |
| HADS | Connect Dutch (Social1) | 0,02 | 0,01 | 0,04 | -0,02 | 0,04 |
| HADS | Connect friends (Social2) | 0,01 | -0,02 | 0,01 | -0,02 | 0,05 |
| HADS | Receive help (Social3) | 0,04 | 0,04 | 0,05 | 0,04 | 0,01 |
| HADS | Help others (Social4) | 0,03 | 0,05 | 0,01 | 0,02 | 0,05 |
| HADS | Expect help (Social5) | 0,00 | -0,04 | -0,01 | 0,02 | 0,01 |
| HADS | Infection (Worry1) | 0,14 | 0,17 | 0,07 | 0,19 | 0,15 |
| HADS | Others infected (Worry2) | 0,10 | 0,08 | 0,14 | 0,03 | 0,11 |
| HADS | Finance (Worry3) | 0,08 | 0,07 | 0,05 | 0,13 | 0,06 |
| HADS | Daily life (Worry4) | 0,07 | 0,08 | 0,07 | 0,09 | 0,04 |
| HADS | Social life (Worry5) | 0,04 | 0,05 | 0,04 | 0,04 | 0,05 |
| Left out (Lonely1) | Isolation (Lonely2) | 0,38 | 0,37 | 0,32 | 0,40 | 0,45 |
| Left out (Lonely1) | Alone (Lonely3) | 0,26 | 0,27 | 0,26 | 0,30 | 0,21 |
| Left out (Lonely1) | Miss company (Lonely4) | 0,04 | 0,01 | 0,03 | 0,02 | 0,08 |
| Left out (Lonely1) | Connect Dutch (Social1) | 0,01 | 0,01 | 0,01 | 0,02 | -0,02 |
| Left out (Lonely1) | Connect friends (Social2) | -0,03 | 0,00 | -0,03 | -0,03 | -0,02 |
| Left out (Lonely1) | Receive help (Social3) | -0,03 | -0,04 | -0,03 | 0,01 | -0,08 |
| Left out (Lonely1) | Help others (Social4) | -0,01 | -0,01 | 0,00 | -0,02 | 0,02 |
| Left out (Lonely1) | Expect help (Social5) | -0,01 | 0,02 | -0,01 | -0,02 | 0,00 |
| Left out (Lonely1) | Infection (Worry1) | 0,02 | -0,03 | 0,04 | 0,00 | 0,02 |
| Left out (Lonely1) | Others infected (Worry2) | 0,01 | 0,03 | 0,00 | 0,01 | 0,01 |
|  |  |  |  |  |  |  |
| **Supplementary Table 1.** Overview of the edge-weights for different network (continued) | | | | | | |
| **From** | **To** | **Total sample**  **(N=4,553)** | **Men**  **<65**  **(N=980)** | **Women <65**  **(N=1,312)** | **Men**  **≥69**  **(N=1,017)** | **Women**  **≥69**  **(N=1,244)** |
| Left out (Lonely1) | Finance (Worry3) | 0,03 | 0,04 | 0,04 | 0,02 | 0,02 |
| Left out (Lonely1) | Daily life (Worry4) | 0,02 | 0,05 | 0,02 | 0,03 | 0,00 |
| Left out (Lonely1) | Social life (Worry5) | 0,02 | 0,00 | 0,03 | 0,03 | 0,02 |
| Isolation (Lonely2) | Alone (Lonely3) | 0,13 | 0,12 | 0,13 | 0,10 | 0,16 |
| Isolation (Lonely2) | Miss company (Lonely4) | 0,11 | 0,13 | 0,12 | 0,14 | 0,07 |
| Isolation (Lonely2) | Connect Dutch (Social1) | 0,01 | 0,01 | 0,01 | 0,00 | 0,02 |
| Isolation (Lonely2) | Connect friends (Social2) | 0,03 | -0,01 | 0,08 | 0,05 | -0,01 |
| Isolation (Lonely2) | Receive help (Social3) | 0,01 | 0,06 | -0,04 | 0,03 | 0,02 |
| Isolation (Lonely2) | Help others (Social4) | -0,05 | -0,06 | -0,06 | -0,04 | -0,02 |
| Isolation (Lonely2) | Expect help (Social5) | -0,01 | -0,03 | 0,01 | -0,01 | -0,01 |
| Isolation (Lonely2) | Infection (Worry1) | 0,04 | 0,11 | 0,03 | -0,01 | 0,03 |
| Isolation (Lonely2) | Others infected (Worry2) | 0,03 | 0,00 | 0,06 | 0,06 | 0,00 |
| Isolation (Lonely2) | Finance (Worry3) | -0,03 | -0,01 | -0,03 | -0,07 | -0,02 |
| Isolation (Lonely2) | Daily life (Worry4) | 0,11 | 0,07 | 0,09 | 0,15 | 0,10 |
| Isolation (Lonely2) | Social life (Worry5) | 0,16 | 0,16 | 0,17 | 0,13 | 0,16 |
| Alone (Lonely3) | Miss company (Lonely4) | 0,30 | 0,31 | 0,34 | 0,29 | 0,27 |
| Alone (Lonely3) | Connect Dutch (Social1) | 0,03 | 0,05 | -0,02 | 0,03 | 0,03 |
| Alone (Lonely3) | Connect friends (Social2) | -0,04 | -0,02 | -0,09 | -0,01 | -0,01 |
| Alone (Lonely3) | Receive help (Social3) | 0,02 | -0,04 | 0,04 | -0,04 | 0,02 |
| Alone (Lonely3) | Help others (Social4) | -0,04 | 0,00 | 0,03 | 0,00 | -0,09 |
| Alone (Lonely3) | Expect help (Social5) | 0,04 | 0,03 | -0,02 | 0,03 | 0,07 |
| Alone (Lonely3) | Infection (Worry1)) | 0,00 | 0,04 | 0,00 | 0,00 | -0,01 |
| Alone (Lonely3) | Others infected (Worry2) | -0,07 | -0,05 | -0,03 | -0,13 | -0,08 |
| Alone (Lonely3) | Finance (Worry3) | 0,02 | 0,01 | 0,05 | 0,03 | -0,02 |
| Alone (Lonely3) | Daily life (Worry4) | -0,01 | 0,00 | 0,00 | 0,01 | -0,03 |
| Alone (Lonely3) | Social life (Worry5) | -0,01 | -0,06 | -0,02 | -0,06 | 0,05 |
| Miss company (Lonely4) | Connect Dutch (Social1) | 0,02 | -0,02 | 0,04 | 0,04 | 0,02 |
| Miss company (Lonely4) | Connect friends (Social2) | -0,01 | 0,04 | -0,03 | -0,06 | 0,03 |
| Miss company (Lonely4) | Receive help (Social3) | -0,01 | -0,03 | -0,01 | 0,00 | 0,00 |
| Miss company (Lonely4) | Help others (Social4) | 0,01 | 0,05 | -0,02 | 0,02 | 0,01 |
| Miss company (Lonely4) | Expect help (Social5) | 0,00 | -0,03 | 0,03 | 0,00 | 0,01 |
| Miss company (Lonely4) | Infection (Worry1) | -0,01 | -0,02 | 0,02 | 0,00 | -0,05 |
| Miss company (Lonely4) | Others infected (Worry2) | -0,01 | -0,06 | -0,01 | 0,02 | 0,01 |
| Miss company (Lonely4) | Finance (Worry3) | -0,01 | -0,10 | 0,04 | -0,04 | 0,02 |
| Miss company (Lonely4) | Daily life (Worry4) | 0,03 | 0,09 | 0,00 | 0,01 | 0,04 |
| Miss company (Lonely4) | Social life (Worry5) | 0,20 | 0,21 | 0,20 | 0,25 | 0,15 |
| Connect Dutch (Social1) | Connect friends (Social2) | 0,31 | 0,31 | 0,31 | 0,30 | 0,32 |
| Connect Dutch (Social1) | Receive help (Social3) | 0,06 | 0,05 | 0,03 | 0,05 | 0,04 |
| Connect Dutch (Social1) | Help others (Social4) | 0,07 | 0,09 | 0,10 | 0,05 | 0,11 |
| Connect Dutch (Social1) | Expect help (Social5) | 0,07 | 0,10 | 0,01 | 0,13 | 0,05 |
| Connect Dutch (Social1) | Infection (Worry1) | 0,07 | 0,07 | 0,07 | 0,08 | 0,03 |
|  |  |  |  |  |  |  |
| **Supplementary Table 1.** Overview of the edge-weights for different network (continued) | | | | | | |
| **From** | **To** | **Total sample**  **(N=4,553)** | **Men**  **<65**  **(N=980)** | **Women <65**  **(N=1,312)** | **Men**  **≥69**  **(N=1,017)** | **Women**  **≥69**  **(N=1,244)** |
| Connect Dutch (Social1) | Others infected (Worry2) | -0,05 | 0,03 | -0,09 | -0,03 | -0,03 |
| Connect Dutch (Social1) | Finance (Worry3) | 0,01 | 0,04 | 0,01 | -0,03 | 0,04 |
| Connect Dutch (Social1) | Daily life (Worry4) | -0,03 | -0,06 | -0,03 | 0,01 | 0,01 |
| Connect Dutch (Social1) | Social life (Worry5) | 0,04 | 0,05 | 0,06 | 0,00 | 0,03 |
| Connect friends (Social2) | Receive help (Social3) | 0,24 | 0,23 | 0,27 | 0,24 | 0,22 |
| Connect friends (Social2) | Help others (Social4) | 0,03 | -0,03 | 0,03 | 0,10 | 0,00 |
| Connect friends (Social2) | Expect help (Social5) | 0,06 | 0,09 | 0,05 | 0,03 | 0,07 |
| Connect friends (Social2) | Infection (Worry1) | -0,02 | -0,05 | 0,03 | -0,04 | -0,03 |
| Connect friends (Social2) | Others infected (Worry2) | 0,06 | 0,06 | 0,02 | 0,07 | 0,07 |
| Connect friends (Social2) | Finance (Worry3) | -0,02 | 0,01 | -0,01 | -0,07 | -0,01 |
| Connect friends (Social2) | Daily life (Worry4) | 0,02 | 0,06 | -0,05 | 0,02 | 0,05 |
| Connect friends (Social2) | Social life (Worry5) | 0,05 | 0,06 | 0,06 | 0,11 | 0,01 |
| Receive help (Social3) | Help others (Social4) | 0,10 | 0,19 | 0,16 | 0,10 | 0,04 |
| Receive help (Social3) | Expect help (Social5) | 0,18 | 0,15 | 0,16 | 0,18 | 0,18 |
| Receive help (Social3) | Infection (Worry1) | 0,03 | 0,04 | -0,01 | 0,04 | 0,04 |
| Receive help (Social3) | Others infected (Worry2) | -0,01 | -0,04 | 0,04 | -0,01 | -0,02 |
| Receive help (Social3) | Finance (Worry3) | -0,01 | 0,00 | -0,03 | 0,02 | 0,02 |
| Receive help (Social3) | Daily life (Worry4) | -0,03 | -0,07 | 0,04 | -0,05 | -0,01 |
| Receive help (Social3) | Social life (Worry5) | 0,02 | 0,06 | -0,04 | 0,00 | 0,02 |
| Help others (Social4) | Expect help (Social5) | 0,34 | 0,39 | 0,36 | 0,31 | 0,31 |
| Help others (Social4) | Infection (Worry1) | -0,07 | -0,10 | -0,10 | -0,02 | -0,01 |
| Help others (Social4) | Others infected (Worry2) | 0,06 | 0,06 | 0,06 | 0,03 | 0,03 |
| Help others (Social4) | Finance (Worry3) | 0,07 | 0,09 | 0,07 | 0,02 | 0,05 |
| Help others (Social4) | Daily life (Worry4) | 0,00 | -0,04 | 0,02 | -0,01 | -0,03 |
| Help others (Social4) | Social life (Worry5) | 0,00 | 0,01 | 0,04 | 0,00 | -0,01 |
| Expect help (Social5) | Infection (Worry1) | 0,01 | 0,07 | -0,01 | 0,00 | 0,00 |
| Expect help (Social5) | Others infected (Worry2) | -0,03 | -0,03 | -0,03 | -0,02 | -0,03 |
| Expect help (Social5) | Finance (Worry3) | -0,05 | -0,05 | -0,04 | -0,02 | -0,07 |
| Expect help (Social5) | Daily life (Worry4) | 0,01 | 0,03 | 0,02 | 0,00 | -0,02 |
| Expect help (Social5) | Social life (Worry5) | 0,02 | 0,01 | 0,02 | 0,01 | 0,03 |
| Infection (Worry1) | Others infected (Worry2) | 0,36 | 0,35 | 0,40 | 0,34 | 0,36 |
| Infection (Worry1) | Finance (Worry3) | 0,06 | 0,09 | 0,06 | 0,08 | 0,05 |
| Infection (Worry1) | Daily life (Worry4) | 0,13 | 0,08 | 0,13 | 0,14 | 0,18 |
| Infection (Worry1) | Social life (Worry5) | 0,05 | 0,06 | 0,00 | 0,07 | 0,06 |
| Others infected (Worry2) | Finance (Worry3) | 0,10 | 0,01 | 0,08 | 0,12 | 0,14 |
| Others infected (Worry2) | Daily life (Worry4) | 0,07 | 0,07 | 0,00 | 0,11 | 0,09 |
| Others infected (Worry2) | Social life (Worry5) | 0,14 | 0,18 | 0,15 | 0,13 | 0,10 |
| Financial worry (Worry3) | Daily life (Worry4) | 0,10 | 0,17 | 0,10 | 0,07 | 0,06 |
| Financial worry (Worry3) | Social life (Worry5) | 0,09 | 0,09 | 0,06 | 0,16 | 0,09 |
| Worry daily life (Worry4) | Social life (Worry5) | 0,31 | 0,31 | 0,34 | 0,27 | 0,34 |

Abbreviations:CESD, Center for Epidemiological Studies Depression scale (depressive symptoms score); HADS, Hospital Anxiety and Depression Scale (anxiety symptoms score)
